# Development and biological characterization of an infectious cDNA clone of NADC34-like PRRSV

**DOI:** 10.3389/fmicb.2024.1359970

**Published:** 2024-05-10

**Authors:** Yafang Lin, Lujia Zhou, Changguang Xiao, Zongjie Li, Ke Liu, Beibei Li, Donghua Shao, Yafeng Qiu, Zhiyong Ma, Jianchao Wei

**Affiliations:** Shanghai Veterinary Research Institute, Chinese Academy of Agricultural Sciences, Shanghai, China

**Keywords:** porcine reproductive and respiratory syndrome virus, NADC34-like, infectious clones, biological characteristics, recombinant virus

## Abstract

**Introduction:**

Porcine Reproductive and Respiratory Syndrome virus (PRRSV) causes high abortion rates in gestating sows and stillbirths, as well as high piglet mortality, seriously jeopardizing the pig industry in China and worldwide.

**Methods:**

In this study, an infectious clone containing the full-length genome of NADC34-like PRRSV was constructed for the first time using reverse genetic techniques. The gene was amplified segmentally onto a plasmid, transfected into BHK-21 cells, and the transfected supernatant was harvested and transfected into PAM cells, which showed classical cytopathic effects (CPE).

**Results:**

The virus rJS-KS/2021 was successfully rescued which could be demonstrated by Western Blot and indirect immunofluorescence assays. Its growth curve was similar to the original strain. Replace the 5’UTR and 3’UTR of rJS-KS/2021 with 5’UTR and 3’UTR of HP-PRRSV (strain SH1) also failed to propagate on MARC-145.

**Discussion:**

In this study, an infectious clone of NADC34-like was constructed by reverse genetics, replacing the UTR and changing the cellular tropism of the virus. These findings provide a solid foundation for studying the recombination of different PRRSVs and the adaption of PRRSVs on MARC-145 in the future.

## 1 Introduction

Porcine reproductive and respiratory syndrome (PRRS) is an acute infectious ailment primarily recognized by clinical manifestations such as late abortion, stillbirth, respiratory distress (interstitial pneumonia), and high mortality rates among pigs, particularly piglets in affected sows (An et al., 2020). This virus causes persistent infections characterized by prolonged viremia, escalated macrophage phagocytosis, and dependence on antibody response (Ouyang et al., 2019). PRRSV is an enveloped and single positive-stranded RNA virus classifying in the family *Arteriviridae*, together with Equine Arteritis Virus, Lactate Dehydrogenase Elevating Virus and Simian Hemorrhagic Fever Virus (Tian, 2017). With an approximate genome length of 15 kilobases (kb), it comprises a minimum of 10 open reading frames (ORFs) and flanking untranslated regions at the 5′ and 3′ ends of the genome (Shanmukhappa and Kapil, 2001; Sun et al., 2018). Secondary structures in the 5’UTR and 3’UTR of PRRSV have important effects on PRRSV replication and transcription (Wootton et al., 2000). ORF1a and ORF1b encode a minimum of 16 non-structural proteins (Nsps) linked to replication. ORF2a, ORF2b, ORF3–5, ORF5a, ORF6, and ORF7 encode eight viral structural proteins (Pujhari and Zakhartchouk., 2016; Liu et al., 2019). Notably, ORF5 has been extensively used in molecular epidemiologic research, as well as for the classification of PRRSV field strains due to its marked genetic variation (Larochelle et al., 2003; Zhu et al., 2021; Yim-Im et al., 2023). Owing to this genetic divergence, PRRSV was divided into two primary genotypes—genotypes 1 and 2, recognized as the European type and the North American type, respectively (Bálint et al., 2021; Wang et al., 2021). Although the clinical symptoms and onset time of these two genotypes are similar, the genome sequences of these two genotypes are only 60% homologous (Li et al., 2022).

PRRSV was first isolated in North America in 1987 and subsequently in the Netherlands, exerting a significant impact on the global swine industry for nearly four decades (Zhao et al., 2018; Wu et al., 2022). Recently, NADC34-like PRRSV has been reported in the United States, China, and Peru and is agreed to be the cause of a large number of clinical abortions (Trevisan et al., 2021; Zhao H. Z. et al., 2022). The pathogenicity of NADC34-like PRRSV in piglets within the United States exhibits a significant degree of variability (Xu et al., 2020). A strain belonging to sublineage 1.5 named NADC34 appear in the United States in 2014 (Kim et al., 2022), which resulting in mass abortion of sows and high mortality of piglets (Xu et al., 2023). It is worth noting that the Nsp2 (non-structural protein 2) gene of the NADC34 strain carries a sustained deletion spanning 100 amino acids (Zhenhua et al., 2018; Xie et al., 2021). The continuous introduction of foreign breeding pigs has led to the recombination of new strains with locally prevalent strains, promoting the diversity of PRRSV (Zhang et al., 2018; Tu et al., 2023). Currently, NADC34-like strains in China account for 11.5 and 28.6% of positives in 2020 and 2021, respectively, demonstrating their pervasive dissemination across at least nine provinces, including Liaoning, Heilongjiang, Fujian, Henan, Sichuan, Jiangsu, Jilin, Hebei, and Shandong (Yuan et al., 2022; Zhao J. et al., 2022). Those indicate the prevalence of NADC34-like PRRSV in China. Intriguingly, The NADC34-like PRRSV strains in Sichuan have different molecular genetic characteristics from other NADC34-like PRRSV strains in China (Xu H. et al., 2022), displaying divergent patterns of amino acid deletion beyond the previously noted 100-amino acid deletion in Nsp2 (Zhang H. et al., 2023). This intricacy contributes to the increased complexity of NADC34-like PRRSV in its native environment (van Geelen et al., 2018). Apart from the documented recombination events, the pathogenicity of NADC34-like PRRSV in the Chinese setting remains elusive. The NADC34-like variant, characterized by substantial mutational alterations, significantly reduced homology, and complex recombination with native Chinese strains, displays a nuanced and intricate landscape for further research (Zhou et al., 2023). Chinese NADC34-like PRRSV exhibits a complex recombination pattern, but as of 2019, it has not recombined with native Chinese strains (Zhao J. et al., 2022). Since 2020, recombination events between NADC34-like viruses and native strains of other lineages have occurred in China. NADC34-like strains have undergone rapid mutations in the Nsp2 region (Xue et al., 2021; Xia et al., 2023). An intricate deletion pattern in the Nsp2 region further distinguishes Chinese NADC34-like strains.

In modern virology, reverse genetic technology is an important tool for the study of RNA viruses, which can produce the genome of the virus from cDNA, and replicate and transcribe it. Technological advancements in PRRSV genome manipulation via reverse genetics have enabled the precise introduction of mutations and deletions in particular genomic areas (Chae et al., 2023). In 1998, the reverse genetic platform of PRRSV-I classical LV strain has been successfully constructed. The full-length LV strain genome was inserted into the pOK12 vector, containing the T7 promoter, as part of the construction strategy. This construction was further transfected into baby hamster kidney-21 (BHK-21) cells. Subsequently, transfected supernatant was harvested and inoculated into MARC-145 cells, resulting in typical cytopathic effects (CPE) following several generations of culture (Meulenberg, 2000). Currently, this system has been widely used in viral genome replication and transcription, virulence, target cell infection, pathogenesis, and immune response, opening up new avenues for molecular research on PRRSV. Several studies have reported that a number of reverse genetic platforms of PRRSV-2 strains have been successfully constructed, including VR-2332, HuN4-F112, and JXwn06 (Zhengda et al., 2022).

In order to further understand the growth characteristics of the virulent strains as well as the pathogenic mechanism, we constructed an infectious clone of NADC34-like PRRSV by reverse genetics to provide a platform for the subsequent study of the vaccine.

## 2 Materials and methods

### 2.1 Cells, strains, and vectors

The MARC-145 and BHK-21 cells were cultured in Dulbecco’s Modified Eagle Medium (DMEM) supplemented with 10% fetal bovine serum (FBS) and antibiotics. Porcine alveolar macrophages (PAMs), the primary target cell for PRRSV, were prepared from lung lavage of specific pathogen-free piglets. PAMs were maintained in Roswell Park Memorial Institute 1,640 medium (RPMI-1640, Gibco, ThermoFisher Scientific, Waltham, MA, United States) supplemented with 10% FBS and antibiotics. The PRRSV isolates [JS-KS/2021 (Zhou et al., 2022)] and the pACYC177 vector were stored at-80°C.

### 2.2 Construction of a full-length cDNA clone

**Figure 1:**
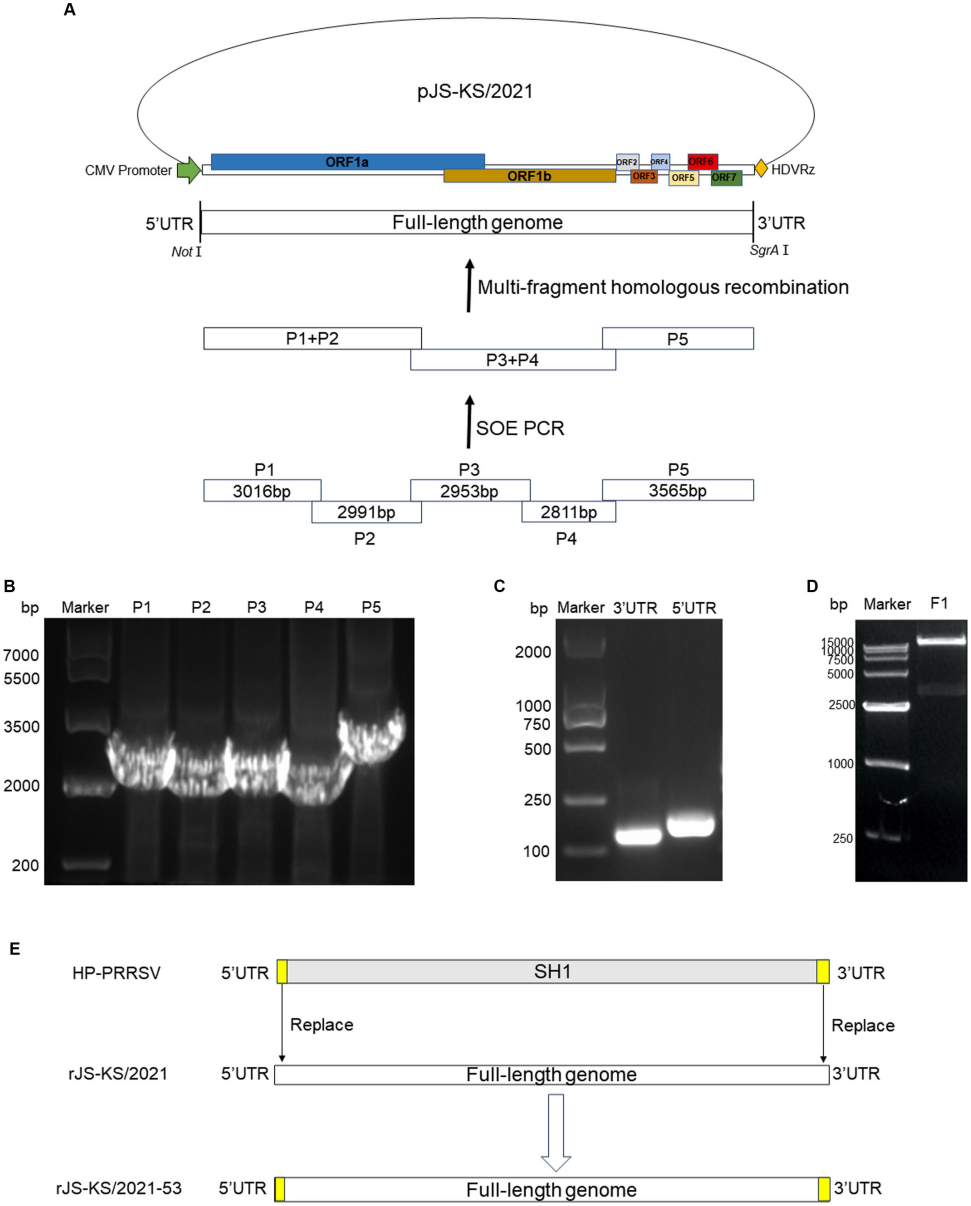
Strategy to construct the full-length cDNA clone of NADC34-Like PRRSV JS-KS/2021 strain. **(A)** Scheme of the NADC34-like PRRSV JS-KS/2021 genome and assembly strategy. The NADC34-like PRRSV JS-KS/2021 genome was divided into five fragments and synthesized. CMV: cytomegalovirus promoter; HDVRz: hepatitis delta virus ribozyme. **(B)** Gel analysis of the five purified cDNA fragments. The P1-P5 fragments were obtained by polymerase chain reaction (PCR) amplification and gel purification. **(C)** The 5’UTR (189 bp) and 3’UTR (155 bp) of HP-PRRSV SH1 were extracted by PCR. **(D)** Infectious clones were verified by *NotI* and *SgrAI* digestion. **(E)** Scheme of pJS-KS/2021–53 by replace the 5’UTR and 3’UTR of rJS-KS/2021 with 5’UTR and 3’UTR of HP-PRRSV strain SH1.

The total RNA from the supernatants of PAM cells at 72 h post-infection (hpi) was used by AG RNAex Pro Reagent (Accurate Biology, Hangzhou, China). The cDNA was synthesized using a M-MLV(H-) Reverse Transcriptase. Specific primer pairs (Table 1) were designed based on the whole genome length to amplify five fragments covering the entire JS-KS/2021 strain genome using a 2× Hieff polymerase chain reaction (PCR) Master Mix (Yeasen Biotechnology, Shanghai, China). The amplification method was performed per the manufacturer’s instructions, and the PCR-amplified fragments were gel-purified. Further, four fragments of the viral genome were fused to produce two new fragments by two-step fusion PCR. Following double digestion of the pACYC177 vector with the appropriate restriction enzymes, the three fragments of the viral genome were inserted into the pACYC177 vector using a homologous recombination kit following the instructions. The scheme used for the construction of a full-length cDNA clone of the JS-KS/2021 strain is indicated in Figure 1A. On the pACYC177 vector, the cytomegalovirus (CMV) promoter was added before the target fragment insertion position, and hepatitis delta virus ribozyme (HDVRz) was added at a subsequent position using primers. Eventually, the completely assembled full-length cDNA clone was sequenced to verify its accuracy and integrity.

**Table 1:** Primers used in this study.

Primer name	Primer sequence (5′-3′)	Location	Length
JS-KS/2021-P1F	ATTTGCGGCCGCATGACGTATAGGTGTTGGCTCTATGCCACGACATTTGTATTGTCGGGAGCTGTGAC	1–41	1
JS-KS/2021-P1R	GTATTTCTCCTTTACCTCTCGGAGGTGCCC	2,945–2,975	
JS-KS/2021-P2F	GGGCACCTCCGAGAGGTAAAGGAGAAATAC	2,945–2,975	2
JS-KS/2021-P2R	TCTCCACAGGAGAAAAAACACACAAAGAAG	5,906–5,936	
JS-KS/2021-P3F	CTTCTTTGTGTGTTTTTTCTCCTGTGGAGA	5,906–5,936	3
JS-KS/2021-P3R	ATCAGGGGCTGGACCTTAAGCATGTCCTCAA	8,829–8,859	
JS-KS/2021-P4F	TTGAGGACATGCTTAAGGTCCAGCCCCTGAT	8,829–8,859	4
JS-KS/2021-P4R	TCTGTCGCGCACGAAACGCGTCATTGTAAT	11,612–11,642	
JS-KS/2021-P5F	ATTACAATGACGCGTTTCGTGCGCGACAGA	11,612–11,642	5
JS-KS/2021-P5R	GAAGGGCTAATGACGCCGGCGAACTTGTTTATTGCAG	14,910–14,940	
SH1-5’UTR-F	ATGACGTATAGGTGTTGGC	1–19	5’UTR
SH1-5’UTR-R	GGTTAAAGGGGTGGAGAGACCG	168–189	
SH1-3’UTR-F	TGGGCTGGCATTCTTTGGCACC	15,171–15,193	3’UTR
SH1-3’UTR-R	AATTACGGCCGCATGGTTC	15,107–15,226	

### 2.3 Construction of recombinant strains with 5’UTR and 3’UTR substitutions

Replace the 5’UTR and 3’UTR of rJS-KS/2021 with 5’UTR and 3’UTR of HP-PRRSV (strain SH1). The 5’UTR and 3’UTR fragments of HP-PRRSV SH1 were amplified by PCR (Figure 1C). The plasmid of the infectious clone constructed was named pJS-KS/2021–53. The scheme of construction could be seen in Figure 1E.

### 2.4 Virus rescue

BHK-21 cells at ~80% confluence in a 24-well culture plate were transfected with 1 μg of a full-length cDNA clone using TurboFect™ transfection reagent (ThermoFisher Scientific, Waltham, MA, United States) according to the manufacturer’s instructions. At 24 h post-transfection (hpt), culture supernatant was harvested to infect PAMs seeded in a 24-well culture plate 1 days ahead. CPE were monitored daily to identify the successful rescue of the virus. Around 4 days post-infection (dpi), the culture supernatant was harvested as F1 virus and stored at-80°C.

### 2.5 Viral growth curve

The confluent PAMs cells in a 24-well culture plate were inoculated with JS-KS/2021, rJS-KS/2021 and rJS-KS/2021–53 at a multiplicity of infection (MOI) of 0. 1. Cells were incubated with virus supernatant diluted with RPMI-1640 for 2 h, then washed twice with 1 × PBS, and cultured with 500 μL RPMI-1640 supplemented with 2% FBS. Virus supernatants were harvested at 0, 12, 24, 36, 48, 60, 72, 84 and 96 hpi for virus titration by TCID_50_. The viral growth curves were created with GraphPad Prism 8.

### 2.6 Indirect immunofluorescence assay

BHK-21 cells were infected with PRRSV at a multiplicity of infection (MOI) of 0.1 and subjected to immunofluorescence assays at 48 hpi. The cells were fixed with 4% paraformaldehyde at room temperature for 30 min and blocked with 5% bovine serum albumin at room temperature for 1 h. For the detection of N protein expression, cells were incubated with an anti-N monoclonal antibody (Lu et al., 2020) at 37°C for 1 h. After three washes, the cell monolayers were stained with FITC-labeled goat anti-mouse IgG. Cell nuclei were counterstained with 4′,6-diamidino-2-phenylindole (DAPI) solution (Solarbio Life Sciences, Beijing, China) for 5 min at room temperature. After extensive washes with 1 × PBS, fluorescent images were captured with epifluorescence microscope.

### 2.7 Western blot

Cellular specimens were harvested and lysed using radio-immunoprecipitation assay buffer comprising 50 mM Tris, pH 7.2, 150 mM NaCl, 1% sodium deoxycholate, and 1% Triton X-100. Following lysate preparation, protein samples were separated on 12% sodium dodecyl sulphate-polyacrylamide gel electrophoresis (SDS-PAGE) gels and were subsequently transferred onto nitrocellulose filter membranes (NC, Cytiva, Washington, United States). The purified blots were incubated with mouse anti-PRRSV N protein (N) antiserum (1,500) at 4°C overnight, followed by treatment with HRP-conjugated goat anti-mouse IgG (1,500 in TBST, TransGen, Beijing, China) at 37°C for 50 min. Signals were detected using the ECL chemiluminescent detection system (Tanon, Shanghai, China) per the manufacturer’s instructions, and resultant images were captured using the Western Blotting imaging system (Tanon, Shanghai, China).

### 2.8 Statistical analysis

The experimental procedures were performed through at least three independent replicates. All acquired data were analyzed using GraphPad Prism 8. The quantified values were indicated as the mean ± standard deviation. Differences were analyzed for statistical significance using two-tailed unpaired t test for two groups or multiple comparison one-way variance (ANOVA) for more than two groups. *p* < 0.05 was considered statistically significant.

## 3 Results

### 3.1 Construction of the full-length infectious cDNA clone

The extraction of viral genomic RNA from the JS-KS/2021 strain followed by reverse transcription to synthesize cDNA, then initiated the generation of a full-length infectious cDNA clone. The resulting viral genome was partitioned into five fragments, denoted as P1-P5, with lengths of 3,016, 2,991, 2,953, 2,811, and 3,565 bp, respectively, as visually presented in Figure 1A. Notably, the observed fragment sizes matched with the anticipated values, as indicated by the congruence displayed in Figure 1B. Following this validation, the fragments were incorporated into a pACYC177 vector, resulting in the successful construction of a full-length cDNA viral plasmid, thereafter referred to as pJS-KS/2021. Its aggregate length was approximately 18.8 kb. Enzymatic digestion of pJS-KS/2021 was verified with *NotI* and *SgrAI* restriction endonucleases, yielding a viral genome of approximately 15 kb (Figure 1D). The results showed that the full-length genome of JS-KS/2021 was successfully inserted into the vector. The efficacy of the reverse genetics system was further validated by the successful rescue of the infective rJS-KS/2021 virus.

### 3.2 The infective rJS-KS/2021 virus was successfully rescued using the reverse genetics system

**Figure 2:**
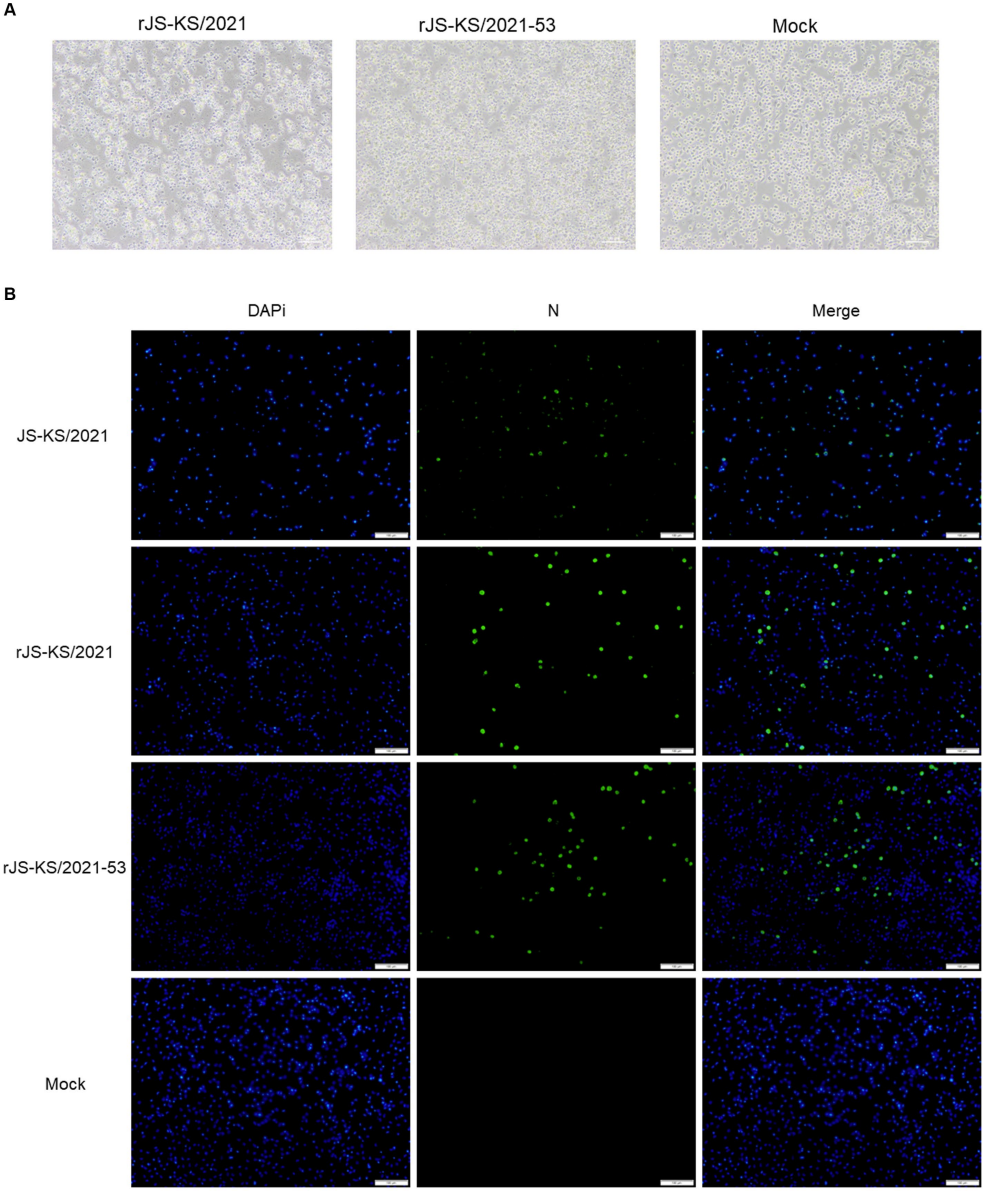
Validation of rJS-KS/2021 and rJS-KS/2021–53. **(A)** Cytopathic effects (CPE) in porcine alveolar macrophages (PAM) cells were observed with rJS-KS/2021 and rJS-KS/2021–53. Classical CPE was observed at 72 hpi compared to negative controls. **(B)** Immunofluorescence staining against the N protein in PAM cells at 48 hpi with JS-KS/2021, rJS-KS/2021 and rJS-KS/2021–53 (MOI = 0.1); mock-infected cells (PAM cells) were used as a negative control. Scale bar =100 μm.

The full-length infectious clone of pJS-KS/2021 was transfected into BHK-21 cells by DNA transfection. At 48 hpi, N protein expression was detected by IFA in cells transfected with rJS-KS/2021 clone (Figure 2B). The N protein expression of the rescued virus and the parental virus was analyzed in PAM cells, the target cells of PRRSV. The findings reveal these viruses could exhibit specific green fluorescence in PAM cells (MOI = 0.1). This finding substantiates the robust expression of the N protein in the target cells by the rJS-KS/2021.

The harvested virus supernatants were further passaged to target cells. MARC-145 cells were used to isolated the virus, but no CPE was observed after incubation for 5 days. After three passages in MARC-145 cells, there was still no CPE. The Reverse Transcription-Polymerase Chain Reaction (RT-PCR) analysis showed that the cell culture was PRRSV-negative. PAMs were then used to isolate the virus with the same sample. At 72hpi, the PAMs showed clustering, detachment, and partial lysis (Figure 2A). In contrast, PAM cells in the blank control group were singly dispersed and morphologically intact. The RT-PCR analysis showed that the cell culture was PRRSV-positive, which was shown to amplify the target bands of the expected size. The sequence of ORF5 of rJS-KS/2021 F3 was verified by sequencing to be the same as ORF5 of the primary virus. The virus was then tried to culture in MARC-145 cells again, but no obvious CPE was observed even after five blind passages. The result indicated that rJS-KS/2021 was unable to propagate in MARC-145 cells.

### 3.3 *In vitro* characterization of the rescued virus rJS-KS/2021

**Figure 3:**
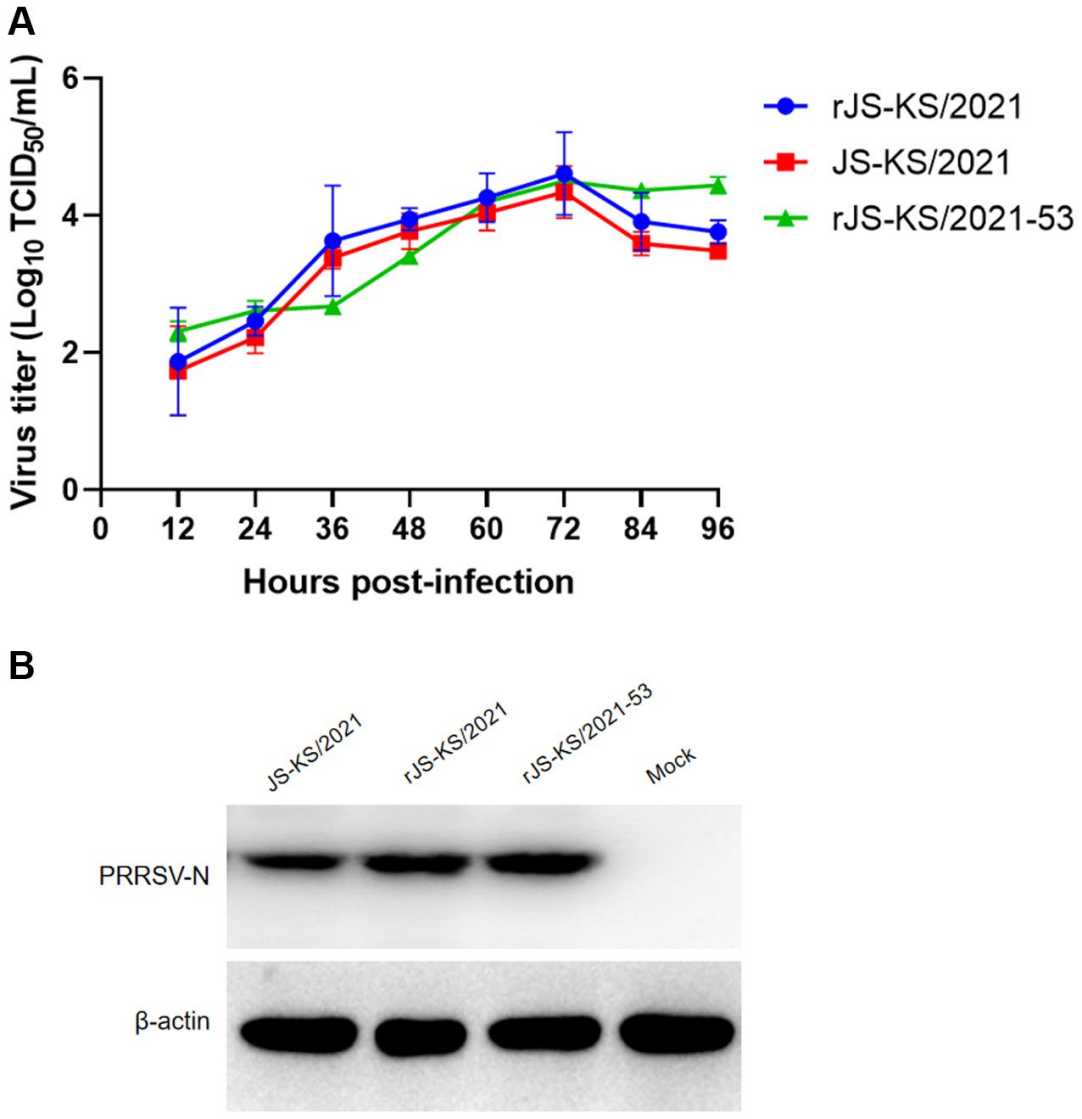
Viral characteristic analysis for rJS-KS/2021 and rJS-KS/2021–53. **(A)** Virus growth kinetics in porcine alveolar macrophages (PAMs). Data are represented by mean ± standard deviation of three independent experiments. **(B)** Western blot analysis of N protein in the virus with JS-KS/2021, rJS-KS/2021 and rJS-KS/2021–53 with specific antibodies against N proteins.

Next, a multiple-step growth curve was performed to characterize the rescued virus. Virus supernatants harvested at 0, 12, 24, 36, 48, 60, 72, 84, and 96 hpi were titrated in PAMs. Based on virus titration results, the parent virus JS-KS/2021 and rescued virus rJS-KS/2021 exhibited similar growth trends and reached their peak titers (10^4.34^ TCID_50_/mL and 10^4.61^ TCID_50_/mL) at 72 hpi, although virus titers of JS-KS/2021 were about 0.3 logs lower than these of rJS-KS/2021 at each time (Figure 3A).

Following infection of PAM cells with rJS-KS/2021, cellular samples were harvested at 24 hpi for subsequent western blot assays. The findings of these assays revealed minimal disparity in N protein expression between the rescued virus and the parental virus, JS-KS/2021, as depicted in Figure 3B.

### 3.4 Characterization of the recombinant virus

The recombinant virus was rescued by DNA transfection of BHK-21 cells. By replacing the 5’UTR and 3’UTR associated with replication capacity, with a view to improving the replication capacity of the virus on PAMs as well as its adaptation at MARC-145. A representative schematic for construction of full-length cDNA clone of rJS-KS/2021–53 was denoted in Figure 1E. Thereafter, the growth characteristics of the recombinant strain were determined. Similarly, the observed band sizes after gel electrophoresis were in accordance with the expected values (Figure 1C). At 72 hpi, typical CPE similar to rJS-KS/2021 were seen on PAMs (Figure 2A), however, no obvious CPE was observed on MARC-145.

The recombinant strain expressed slightly more N protein than rJS-KS/2021(Figure 2B), and we obtained the same result when virus supernatants were harvestred from infected PAMs 24 hpi for western blot assay. This shows that the recombinant strain has a slightly higher replication ability than rJS-KS/2021 at the early stage of viral infection. But when PAMs cells were infected at MOI = 0.1 for the growth curve analysis, the recombinant strain replicates faster at the early stage and reaches a high titer at 72 hpi compared to rJS-KS/2021. The viral titer after 72 hpi tends to stabilize, and higher than rJS-KS/2021. These results demonstrate that replacing the 5’UTR and 3’UTR of HP-PRRSV improves the replication ability of the recombinant strain, but does not change the fitness of the recombinant strain on MARC-145.

## 4 Discussion

NADC34-like PRRSV was reported for the first time in 2017 in the pig farms of Liaoning Province in China (An et al., 2020). The virus was identified as the causal agent of the symptoms of late abortion, stillbirth, dry or weak fetuses, respiratory distress (interstitial pneumonia) and high mortality in pigs of different ages, especially piglets (Xu Y. et al., 2022). Another way PRRSV affects pigs is through an increased incidence of secondary bacterial infections, including Haemophilus parvum, Pseudomonas multiforme, and Pseudomonas pleuropneumoniae (Bao and Li, 2021; Zhang W. et al., 2023). To date, infections and outbreaks caused by PRRSV have not been well controlled and prevented and thus remain prevalent in numerous countries, and particularly, the emergence of mutants or new strains occasionally causes outbreaks and re-emergence of the disease (Ma et al., 2022). The continuous introduction of foreign breeds has resulted in the recombination of new strains with locally prevalent strains, promoting the diversity of PRRSV (Xia et al., 2023). However, the pathogenicity of NADC34-like PRRSV in China remains elusive (Rawal et al., 2023).

The detection rate of NADC34-Like PRRSV has been on the rise in recent years. In 2017–2019, the PRRSV positivity rate was less than 3%, which gradually increased to 11.5% in 2020. It finally reaches a staggering 28.6% positivity rate. It can be seen that NADC34-Like and NADC30-Like (35.4%) together with highly pathogenic (HP)-PRRSV (31.2%) have become the major strains in some areas of China and are becoming more and more prevalent (Zhao J. et al., 2022). The NADC34 strain has been mainly isolated in the northern provinces of Heilongjiang, Henan, Shandong, Hebei, Jilin, Jiangsu, and Liaoning. Thus, the southern provinces must closely monitor the epidemic trend of this strain (Kappes and Faaberg., 2015; Zhao J. et al., 2022). The NADC34-like PRRSV strains in Sichuan differ from other NADC34-like PRRSV strains in China in terms of molecular genetic characteristics (Xu et al., 2020). The variation in pathogenicity across various NADC34 may be attributed to the different recombination patterns with different PRRSV strains (Nanhua et al., 2013; Pamornchainavakul et al., 2022). Recombination is a common phenomenon among PRRSV isolates and a significant genetic variation that is observed in PRRSV (Wang et al., 2018; Yu et al., 2020; Zhou et al., 2023). A distinct pattern of amino acid (aa) deletion was observed in addition to recombination events, in contrast to the 100-aa deletion in NSP2 (Xie et al., 2022). Consequently, in the wild, NADC34-like PRRSV is more complex (Bao and Li, 2021). To date, there have been no reports of experimental infections of NADC34 in sows and gilts.

Within the realm of molecular biology, reverse genetics is significant techniques, particularly in the fundamental investigation of RNA viruses. Consequently, this technology is extremely important for the advancement of molecular research on PRRSV (Chae et al., 2023). The conventional approach to constructing infectious cDNA molecular clone entails segmenting the genome according to the distribution of restriction sites, amplifying the corresponding viral genome segments, and ligating them with appropriate vectors. This is responsible for controlling the complete cDNA of the PRRSV genome under the influence of robust transcription promoters. Frequently employed promoters encompass bacteriophage T7, T3, SP6, and CMV promoters. Initially, the full-length genome is synthesized, transcribed *in vitro* (T7, T3, and SP6 promoters), and transfected, and subsequently, infectious virus particles are regenerated. It is crucial to note that mutations in fragment nucleotides during the construction and reverse transcription process can impede the successful recovery of infectious clones. Since 1998, infectious clones have been successfully produced using the LV strain as a template, causing successive acquisition of multiple infectious clones (Meulenberg, 2000; Shin et al., 2022). In recent endeavors, numerous infectious clones of both classical-and HP-PRRSV have been carefully engineered in the scientific community of China. Reverse genetic systems have become an important tool in the field of picornavirus research, particularly supporting the investigation of NADC34-like PRRSV. This advanced technology provides a strong technical basis for examining diverse facets such as viral replication, pathogenic mechanisms, virulence genes, molecular details of viral-host interactions, *in vivo* functionality of viral proteins, the creation of live viral vectors, and the development of recombinant genetically engineered vaccines (Zhengda et al., 2022).

CMV is generally recognized as the most powerful promoter of eukaryotic gene expression. Compared with H1/U6 promoter, which is a prokaryotic promoter and has very low initiation efficiency in eukaryotic cells, CMV is a triple-type promoter, which can be initiated in both short and long lengths, and can initiate sequences with PolyA tails. Generally, the CDS region of a gene is inserted downstream of the CMV promoter, and the CMV is responsible for initiating the expression of the gene, so as to increase the expression of the gene. It is most used in the construction of adenovirus and lentivirus overexpression vectors. In contrast to the high specificity of the T7 promoter, which can only be initiated by T7 polymerase binding, the CMV promoter can be initiated by a wide range of polymerases, including *E.coli* RNA polymerase and T7 RNA polymerase. In order to produce stable and generous viruses, we constructed a full-length infectious cDNA clone (Figure 1A), rescued the virus of the NADC34-like strain, JS-KS/2021. In this study, constructing an infectious clone with a low copy plasmid and placing the CMV promoter in front of the viral genome allowed strict control of viral genome replication and reduced toxicity to cells. The rescued virus rJS-KS/2021 was able to propagate well on PAMs cells (Figure 2A). The biological characteristics of the rescued virus was further evaluated (Figures 2, 3). The results show that rJS-KS/2021 was able to express more N proteins than the parental strain JS-KS/2021. In the analysis of growth curves, rJS-KS/2021 had similar growth characteristics to the parental strain JS-KS/2021 and the viral titer was about 0.3 logs higher than that of the parental strain at each time point.

It is known that the 5′ and 3’ UTRs of PRRSV both play critical roles in replication, subgenomic RNA transcription, and infectivity (Verheije et al., 2002; Yin et al., 2013). Shanmukhappa et al. (2007) firstly indicated that 3’UTR RNA of PRRSV is responsible for the interaction with CD151 receptor. CD151 has been identified as another important PRRSV cellular receptor. CD151 is expressed in all PRRSV permissive cell lines, including PAMs, MA-104, MARC-145, Vero, COS-7 and SJPG cells (Shanmukhappa et al., 2007; Provost et al., 2012), while the PRRSV nonpermissive cell lines, such as BHK-21 and MDBK cells, do not express CD151 protein (Shanmukhappa et al., 2007). In 2007, Shanmukhappa et al. (2007) reported that they identified CD151 plays a significant role during PRRSV infection of target cells by a serial of experiments. By the transfection of CD151 clone into BHK-21, the non-susceptible cells became permissive to PRRSV infection. In our study, in view of the replication looping of the 5’UTR and 3’UTR of PRRSV, replace the 5’UTR and 3’UTR of rJS-KS/2021 with the 5’UTR and 3’UTR of HP-PRRSV (strain SH1). The recombinant strain rJS-KS/2021–53 could stably express more N proteins in the early stage of viral infection, which demonstrated by indirect immunofluorescence and western blot assays (Figures 2B, 3B). The growth curve can also support this result (Figure 3A). However, the viral titer after 72 hpi tends to stabilize, higher than the rescued virus rJS-KS/2021 and the parental virus JS-KS/2021. Most importantly, the recombinant strain infected PAMs well, but also failed to propagate on MARC-145. These infectious clones constructed of NADC34-like PRRSV prove reverse genetics becoming more and more important techniques in study of PRRSVs. Moreover, provide a platform for further research on cellular tropism of PRRSV through replacement and mutation.

## 5 Conclusion

In the present study, we successfully established a reverse genetics system for NADC34-Like PRRSV. Next, we demonstrated that a full-length cDNA clone was able to replicate and infect PAM cells. Also, we replaced the 5’UTR and 3’UTR of rJS-KS/2021 with these of HP-PRRSV (strain SH1). Unfortunately, rJS-KS/2021 and the recombinant virus could not propagate on MARC-145. We believe that this rJS-KS/2021 infectious clone and the information generated in the present study will make a significant contribution to future studies on basic viral biology and to the development of NADC34-Like PRRSV control measures.

## Data availability statement

The raw data supporting the conclusions of this article will be made available by the authors, without undue reservation.

## Author contributions

YL: Formal analysis, Methodology, Validation, Writing – original draft. LZ: Formal analysis, Investigation, Methodology, Validation, Writing – original draft. CX: Formal analysis, Visualization, Writing – original draft, Writing – review & editing. ZL: Investigation, Validation, Writing – review & editing. KL: Investigation, Validation, Writing – review & editing. BL: Investigation, Validation, Writing – review & editing. DS: Investigation, Validation, Writing – review & editing. YQ: Investigation, Validation, Writing – review & editing. ZM: Conceptualization, Funding acquisition, Project administration, Writing – review & editing. JW: Conceptualization, Formal analysis, Funding acquisition, Methodology, Writing – review & editing.

## References

[ref1] An T. Q. Li J. N. Su C. M. Yoo D. (2020). Molecular and cellular mechanisms for PRRSV pathogenesis and host response to infection. Virus Res. 286:197980. doi: 10.1016/j.virusres.2020.197980 32311386 PMC7165118

[ref2] Bálint Á. Molnár T. Kecskeméti S. Kulcsár G. Soós T. Szabó P. M.. (2021). Genetic variability of PRRSV vaccine strains used in the National Eradication Programme, Hungary. Vaccine 9:849. doi: 10.3390/vaccines9080849, PMID: 34451974 PMC8402617

[ref3] Bao H. Li X. (2021). Emergence and spread of NADC34-like PRRSV in China. Transbound. Emerg. Dis. 68, 3005–3008. doi: 10.1111/tbed.14316 34492162

[ref4] Chae H. Roh H. S. Jo Y. M. Kim W. G. Chae J. B. Shin S. U.. (2023). Development of a one-step reverse transcription-quantitative polymerase chain reaction assay for the detection of porcine reproductive and respiratory syndrome virus. PLoS One 18:e0293042. doi: 10.1371/journal.pone.0293042, PMID: 37844073 PMC10578580

[ref6] Kappes M. A. Faaberg K. S. (2015). PRRSV structure, replication and recombination: origin of phenotype and genotype diversity. Virology 479, 475–486. doi: 10.1016/j.virol.2015.02.012 25759097 PMC7111637

[ref7] Kim S. C. Moon S. H. Jeong C. G. Park G. S. Park J. Y. Jeoung H. Y.. (2022). Whole-genome sequencing and genetic characteristics of representative porcine reproductive and respiratory syndrome virus (PRRSV) isolates in Korea. Virol. J. 19:66. doi: 10.1186/s12985-022-01790-6 35410421 PMC8996673

[ref8] Larochelle R. D'Allaire S. Magar R. (2003). Molecular epidemiology of porcine reproductive and respiratory syndrome virus (PRRSV) in Québec. Virus Res. 96, 3–14. doi: 10.1016/S0168-1702(03)00168-0, PMID: 12951261

[ref9] Li Y. Jiao D. Jing Y. He Y. Han W. Li Z.. (2022). Genetic characterization and pathogenicity of a novel recombinant PRRSV from lineage 1, 8 and 3 in China failed to infect MARC-145 cells. Microb. Pathog. 165:105469. doi: 10.1016/j.micpath.2022.105469 35271985

[ref10] Liu J. Wei C. Lin Z. Xia W. Ma Y. Dai A.. (2019). Full genome sequence analysis of a 1-7-4-like PRRSV strain in Fujian Province, China. PeerJ 7:e7859. doi: 10.7717/peerj.7859, PMID: 31637126 PMC6800524

[ref001] Lu Y. Zhang Y. Xiang X. Sharma M. Liu K. Wei J.. (2020). Notch signaling contributes to the expression of inflammatory cytokines induced by highly pathogenic porcine reproductive and respiratory syndrome virus (HP-PRRSV) infection in porcine alveolar macrophages. Dev. Comp. Immunol. 108:103690. doi: 10.1016/j.dci.2020.103690 32222356 PMC7765342

[ref11] Ma X. Wang P. Zhang R. Zhao Y. Wu Y. Luo C.. (2022). A NADC30-like PRRSV causes serious intestinal infections and tropism in piglets. Vet. Microbiol. 268:109397. doi: 10.1016/j.vetmic.2022.109397, PMID: 35364367

[ref12] Meulenberg J. (2000). PRRSV, the virus. Vet. Res. 31, 11–21. doi: 10.1051/vetres:2000103 10726635

[ref13] Nanhua C. Xiuling Y. Lilin W. Jiajun W. Zhi Z. Jianqiang N.. (2013). Two natural recombinant highly pathogenic porcine reproductive and respiratory syndrome viruses with different pathogenicities. Virus Genes 46, 473–478. doi: 10.1007/s11262-013-0892-4, PMID: 23430712

[ref14] Ouyang T. Zhang X. Liu X. Ren L. (2019). Co-infection of swine with porcine circovirus type 2 and other swine viruses. Viruses 11:185. doi: 10.3390/v11020185 30795620 PMC6410029

[ref15] Pamornchainavakul N. Kikuti M. Paploski I. A. D. Makau D. N. Rovira A. Corzo C. A.. (2022). Measuring how recombination re-shapes the evolutionary history of PRRSV-2: a genome-based Phylodynamic analysis of the emergence of a novel PRRSV-2 variant. Front Vet Sci 9:846904. doi: 10.3389/fvets.2022.846904, PMID: 35400102 PMC8990846

[ref16] Provost C. Jia J. J. Music N. Lévesque C. Lebel M. del Castillo J. R.. (2012). Identification of a new cell line permissive to porcine reproductive and respiratory syndrome virus infection and replication which is phenotypically distinct from MARC-145 cell line. Virol. J. 9:267. doi: 10.1186/1743-422X-9-267 23148668 PMC3546013

[ref17] Pujhari S. Zakhartchouk A. N. (2016). Porcine reproductive and respiratory syndrome virus envelope (E) protein interacts with mitochondrial proteins and induces apoptosis. Arch. Virol. 161, 1821–1830. doi: 10.1007/s00705-016-2845-4, PMID: 27068165

[ref18] Rawal G. Almeida M. N. Gauger P. C. Zimmerman J. J. Ye F. Rademacher C. J.. (2023). In vivo and in vitro characterization of the recently emergent PRRSV 1–4-4 L1C variant (L1C.5) in comparison with other PRRSV-2 lineage 1 isolates. Viruses 15:2233. doi: 10.3390/v15112233 38005910 PMC10674456

[ref19] Shanmukhappa K. Kapil S. (2001). Cloning and identification of MARC-145 cell proteins binding to 3'UTR and partial nucleoprotein gene of porcine reproductive and respiratory syndrome virus. Adv. Exp. Med. Biol. 494, 641–646. doi: 10.1007/978-1-4615-1325-4_95 11774539

[ref20] Shanmukhappa K. Kim J. K. Kapil S. (2007). Role of CD151, a tetraspanin, in porcine reproductive and respiratory syndrome virus infection. Virol. J. 4:62. doi: 10.1186/1743-422X-4-62 17572908 PMC1906853

[ref21] Shin G. E. Park J. Y. Lee K. K. Ko M. K. Ku B. K. Park C. K.. (2022). Genetic diversity of porcine reproductive and respiratory syndrome virus and evaluation of three one-step real-time RT-PCR assays in Korea. BMC Vet. Res. 18:327. doi: 10.1186/s12917-022-03407-0 36042510 PMC9429472

[ref22] Sun Y. F. Zhou L. Bian T. Tian X.-X. Ren W.-K. Lu C.. (2018). Efficacy evaluation of two commercial modified-live virus vaccines against a novel recombinant type 2 porcine reproductive and respiratory syndrome virus. Vet. Microbiol. 216, 176–182. doi: 10.1016/j.vetmic.2018.02.016, PMID: 29519513

[ref23] Tian K. (2017). NADC30-like porcine reproductive and respiratory syndrome in China. Open Virol. J. 11, 59–65. doi: 10.2174/1874357901711010059 28839505 PMC5543618

[ref24] Trevisan G. Li G. Moura C. A. A. Coleman K. Thomas P. Zhang J.. (2021). Complete coding genome sequence of a novel porcine reproductive and respiratory syndrome virus 2 restriction fragment length polymorphism 1-4-4 lineage 1C variant identified in Iowa, USA. Microbiol. Resour. Announc. 10:e0044821. doi: 10.1128/MRA.00448-21, PMID: 34042485 PMC8213044

[ref25] Tu T. Pang M. Jiang D. Zhou Y. Wu X. Yao X.. (2023). Development of a real-time TaqMan RT-PCR assay for the detection of NADC34-like porcine reproductive and respiratory syndrome virus. Vet. Sci. 10:279. doi: 10.3390/vetsci10040279, PMID: 37104434 PMC10141196

[ref26] van Geelen A. G. M. Anderson T. K. Lager K. M. Das P. B. Otis N. J. Montiel N. A.. (2018). Porcine reproductive and respiratory disease virus: evolution and recombination yields distinct ORF5 RFLP 1-7-4 viruses with individual pathogenicity. Virology 513, 168–179. doi: 10.1016/j.virol.2017.10.002, PMID: 29096159

[ref27] Verheije M. H. Olsthoorn R. C. Kroese M. V. Rottier P. J. Meulenberg J. J. (2002). Kissing interaction between 3′ noncoding and coding sequences is essential for porcine arterivirus RNA replication. J. Virol. 76, 1521–1526. doi: 10.1128/JVI.76.3.1521-1526.2002, PMID: 11773426 PMC135790

[ref28] Wang H. M. Liu Y. G. Tang Y. D. Liu T. X. Zheng L. L. Wang T. Y.. (2018). A natural recombinant PRRSV between HP-PRRSV JXA1-like and NADC30-like strains. Transbound. Emerg. Dis. 65, 1078–1086. doi: 10.1111/tbed.12890, PMID: 29520988

[ref29] Wang S. Liu Y. Yu L. Liang T. Zhang P. Dong J.. (2021). A strain of highly pathogenic porcine reproductive and respiratory syndrome virus: genomic characterization, pathogenicity, and construction of an infectious full-length cDNA clone. Arch. Virol. 166, 3127–3141. doi: 10.1007/s00705-021-05212-w, PMID: 34529151

[ref30] Wootton S. Yoo D. Rogan D. (2000). Full-length sequence of a Canadian porcine reproductive and respiratory syndrome virus (PRRSV) isolate. Arch. Virol. 145, 2297–2323. doi: 10.1007/s007050070022, PMID: 11205119 PMC7086845

[ref31] Wu Y. Peng O. Xu Q. Li Q. Li W. Lin L.. (2022). Characterization and pathogenicity of two novel PRRSVs recombined by NADC30-like and NADC34-like strains in China. Viruses 14:2174. doi: 10.3390/v14102174 36298730 PMC9607012

[ref32] Xia Y. Zhang T. Gong D. Qi J. Jiang S. Yang H.. (2023). Recombination and mutation in a new HP-PRRSV strain (SD2020) from China. Viruses 15:165. doi: 10.3390/v15010165 36680205 PMC9864264

[ref33] Xie C. Z. Tao Y. M. Ha Z. Zhang P. Zhang Y. Zhang H.. (2022). Characterization of a new NSP2-deletion NADC34-like porcine reproductive and respiratory syndrome virus in China. Res. Vet. Sci. 152, 212–218. doi: 10.1016/j.rvsc.2022.08.001 35998397

[ref34] Xie C. Z. Wang Z. Ha Z. Zhang Y. Xie Y. B. Zhang H.. (2021). Genetic characterization of a new NSP2-deletion porcine reproductive and respiratory syndrome virus in China. Microb. Pathog. 150:104729. doi: 10.1016/j.micpath.2021.104729 33429053

[ref35] Xu H. Li C. Gong B. Li W. Guo Z. Sun Q.. (2023). Protective efficacy of a candidate live-attenuated vaccine derived from the SD-R strain against NADC34-like porcine reproductive and respiratory syndrome virus. Vaccine 11:1349. doi: 10.3390/vaccines11081349, PMID: 37631917 PMC10459522

[ref36] Xu H. Li C. Li W. Zhao J. Gong B. Sun Q.. (2022). Novel characteristics of Chinese NADC34-like PRRSV during 2020-2021. Transbound. Emerg. Dis. 69, e3215–e3224. doi: 10.1111/tbed.14485, PMID: 35182461

[ref37] Xu H. Song S. Zhao J. Leng C. Fu J. Li C.. (2020). A potential endemic strain in China: NADC34-like porcine reproductive and respiratory syndrome virus. Transbound. Emerg. Dis. 67, 1730–1738. doi: 10.1111/tbed.13508 32037673

[ref38] Xu Y. Ye M. Sun S. Cao Q. Luo J. Wang Y.. (2022). CD163-expressing porcine macrophages support NADC30-like and NADC34-like PRRSV infections. Viruses 14:2056. doi: 10.3390/v14092056 36146862 PMC9505768

[ref39] Xue R. X. Sun S. F. Li Y. G. Wang M. L. Wang G. S. Li Y. J.. (2021). Diversity of porcine reproductive and respiratory syndrome virus in Shandong, China. Acta Virol. 65, 303–306. doi: 10.4149/av_2021_305 34565158

[ref40] Yim-Im W. Anderson T. K. Paploski I. A. VanderWaal K. Gauger P. Krueger K.. (2023). Refining PRRSV-2 genetic classification based on global ORF5 sequences and investigation of their geographic distributions and temporal changes. Microbiol. Spectr. 11:e0291623. doi: 10.1128/spectrum.02916-23 37933982 PMC10848785

[ref41] Yin Y. Liu C. Liu P. Yao H. Wei Z. Lu J.. (2013). Conserved nucleotides in the terminus of the 3' UTR region are important for the replication and infectivity of porcine reproductive and respiratory syndrome virus. Arch. Virol. 158, 1719–1732. doi: 10.1007/s00705-013-1661-3, PMID: 23512575

[ref42] Yu F. Yan Y. Shi M. Liu H. Z. Zhang H. L. Yang Y. B.. (2020). Phylogenetics, genomic recombination, and NSP2 polymorphic patterns of porcine reproductive and respiratory syndrome virus in China and the United States in 2014-2018. J. Virol. 94:e01813-19. doi: 10.1128/JVI.01813-19 31896589 PMC7158704

[ref43] Yuan L. Zhu Z. Fan J. Liu P. Li Y. Li Q.. (2022). High pathogenicity of a Chinese NADC34-like PRRSV on pigs. Microbiol. Spectr. 10:e0154122. doi: 10.1128/spectrum.01541-22 35766496 PMC9431460

[ref44] Zhang H. Luo Q. Zheng Y. Sha H. Li G. Kong W.. (2023). Genetic variability and recombination of the NSP2 gene of PRRSV-2 strains in China from 1996 to 2021. Vet. Sci. 10:325. doi: 10.3390/vetsci10050325 37235408 PMC10224145

[ref45] Zhang W. Ma W. Pan Y. Wang X. Wang M. Zhang H.. (2023). Characterization of Rongchang piglets after infection with type 2 porcine reproductive and respiratory syndrome virus strains differing in pathogenicity. Front. Microbiol. 14:1283039. doi: 10.3389/fmicb.2023.1283039, PMID: 37920268 PMC10618352

[ref46] Zhang H.-L. Zhang W.-L. Xiang L.-R. Leng C.-L. Tian Z.-J. Tang Y.-D.. (2018). Emergence of novel porcine reproductive and respiratory syndrome viruses (ORF5 RFLP 1-7-4 viruses) in China. Vet. Microbiol. 222, 105–108. doi: 10.1016/j.vetmic.2018.06.017, PMID: 30080663

[ref47] Zhao K. Gao J. C. Xiong J. Y. Guo J. C. Yang Y. B. Jiang C. G.. (2018). Two residues in NSP9 contribute to the enhanced replication and pathogenicity of highly pathogenic porcine reproductive and respiratory syndrome virus. J. Virol. 92:e02209-17. doi: 10.1128/JVI.02209-17, PMID: 29321316 PMC5972891

[ref48] Zhao H. Z. Wang F. X. Han X. Y. Guo H. Liu C. Y. Hou L. N.. (2022). Recent advances in the study of NADC34-like porcine reproductive and respiratory syndrome virus in China. Front. Microbiol. 13:950402. doi: 10.3389/fmicb.2022.950402 35935186 PMC9354828

[ref49] Zhao J. Xu L. Xu Z. Deng H. Li F. Sun X.. (2022). Emergence and spread of NADC34-like PRRSV in Southwest China. Transbound. Emerg. Dis. 69, e3416–e3424. doi: 10.1111/tbed.14463, PMID: 35090082

[ref50] Zhengda C. Jinxia C. Liwei L. Jiachen L. Wu T. Yanjun Z.. (2022). A rescued NADC30-like virus by reverse genetic manipulation exhibits moderate virulence and a promising application perspective. Virus Res. 316:198801. doi: 10.1016/j.virusres.2022 35550390

[ref51] Zhenhua G. Xin-Xin C. Rui L. Songlin Q. Gaiping Z. (2018). The prevalent status and genetic diversity of porcine reproductive and respiratory syndrome virus in China: a molecular epidemiological perspective. Virol. J. 15:2. doi: 10.1186/s12985-017-0910-6 29301547 PMC5753475

[ref52] Zhou L. Yang Y. Xia Q. Guan Z. Zhang J. Li B.. (2022). Genetic characterization of porcine reproductive and respiratory syndrome virus from eastern China during 2017-2022. Front. Microbiol. 13:971817. doi: 10.3389/fmicb.2022.971817, PMID: 36312912 PMC9606797

[ref53] Zhou L. Yu J. Zhou J. Long Y. Xiao L. Fan Y.. (2023). A novel NADC34-like porcine reproductive and respiratory syndrome virus 2 with complex genome recombination is highly pathogenic to piglets. Infect. Genet. Evol. 112:105436. doi: 10.1016/j.meegid.2023.105436, PMID: 37094706

[ref54] Zhu Z. Yuan L. Hu D. Lian Z. Yao X. Liu P.. (2021). Isolation and genomic characterization of a Chinese NADC34-like PRRSV isolated from Jiangsu province. Transbound. Emerg. Dis. 69, e1015–e1027. doi: 10.1111/tbed.14392 34786872

